# Characteristics of Adults With Non‐Hospitalized Severe Respiratory Illness: Findings From a COVID‐19 Vaccine Effectiveness Evaluation in Kenya, 2022–2023

**DOI:** 10.1111/irv.70145

**Published:** 2025-08-06

**Authors:** Radhika Gharpure, Young M. Yoo, Bryan O. Nyawanda, Raphael O. Anyango, Brian O. Onyando, Sidney Ogolla, Billy Ogwel, Eric Osoro, Philip Ngere, Samuel Kadivane, Nzisa Liku, Eva Leidman, Gideon O. Emukule, Richard Omore, Kathryn E. Lafond

**Affiliations:** ^1^ U.S. Centers for Disease Control and Prevention Atlanta Georgia USA; ^2^ Kenya Medical Research Institute Center for Global Health Research Kisumu Kenya; ^3^ Washington State University Global Health Kenya Nairobi Kenya; ^4^ Paul G. Allen School for Global Health Washington State University Pullman USA; ^5^ Ministry of Health Division of Disease Surveillance and Response Nairobi Kenya; ^6^ US Centers for Disease Control and Prevention Nairobi Kenya

**Keywords:** burden of disease, hospitalization, Kenya, outpatients, respiratory viruses

## Abstract

Studies suggest the burden of non‐hospitalized severe respiratory illness might be substantial in Kenya. Using data from a Kenya COVID‐19 vaccine effectiveness evaluation, we compared characteristics of patients aged ≥12 years who were hospitalized with severe respiratory illness to outpatients who were referred for hospitalization and declined (non‐hospitalized). Symptom presentation and lung radiograph findings were similar among both groups, and patients in both were diagnosed with critical conditions, including acute respiratory distress syndrome (12% hospitalized; 4% non‐hospitalized) and sepsis (10% both). Findings underscore the importance of including non‐hospitalized severe illness when estimating the burden of disease for respiratory viruses.

## 1 Introduction

Estimating the disease burden caused by vaccine‐preventable respiratory viruses such as influenza virus, severe acute respiratory syndrome coronavirus 2 (SARS‐CoV‐2), and respiratory syncytial virus (RSV) can provide valuable evidence to guide planning, investment, and evaluation for immunization programs. Standardized case definitions for severe acute respiratory infection (SARI) [1], used for syndromic surveillance of respiratory hospitalizations at sentinel healthcare facilities, are commonly used to estimate the burden of severe respiratory disease [2]; however, these definitions can have varying sensitivity and specificity by age [3, 4, 5] and alone cannot measure the burden of non‐hospitalized severe respiratory disease.

Multiple studies have suggested that the burden of non‐hospitalized severe respiratory disease might be substantial in Kenya [6, 7, 8]. A 2009–2012 analysis from Western Kenya indicated that the rates of non‐medically attended severe respiratory illnesses associated with influenza and RSV were higher than the rates of medically attended cases for both viruses [6]. Similarly, a national analysis from 2012 to 2014 indicated that the annual mean rate of non‐hospitalized influenza‐associated cases in Kenya (847.1 per 100,000 persons) was 3.7 times higher than the rate of hospitalized influenza‐associated cases [7]. A 2018 healthcare utilization survey across four Kenyan counties suggested that only 1 in 5 (20.8%) patients with severe pneumonia were hospitalized [8]. Collectively, these findings indicate a need to further evaluate non‐hospitalized severe respiratory illness in the Kenyan context.

In this analysis, we examined characteristics of patients with severe respiratory illness who presented at 20 referral health facilities in Kenya from July 2022 to August 2023, comparing characteristics of hospitalized patients with those of non‐hospitalized patients (referred for hospitalization in an outpatient clinic but declined).

## 2 Methods

This study was a secondary analysis of data collected for a COVID‐19 vaccine effectiveness evaluation in Kenya [9]. Briefly, the evaluation enrolled patients aged ≥12 years at 20 referral health facilities during July 2022–August 2023 to estimate COVID‐19 vaccine effectiveness using a test‐negative case–control design adapted from World Health Organization (WHO) guidance [10]. Patients were enrolled if they met the case definition for severe respiratory illness (SRI), an expanded case definition from the traditional SARI surveillance case definition; this included hospitalization or a clinician's recommendation for hospitalization for acute respiratory illness (onset within the last 14 days) and meeting one of the following clinical criteria: (1) at least two compatible symptoms, defined as cough, fever (reported or measured with a temperature of ≥38 °C), chills, rigors, myalgia, headache, sore throat, fatigue, nasal congestion, runny nose, or loss of taste or smell or (2) pneumonia (based on clinical diagnosis or radiographic evidence).

Patients were enrolled in each facility's inpatient ward (IPD) if hospitalized, or in the outpatient clinic (OPD) if recommended for hospitalization but not hospitalized. At enrollment, evaluation staff collected information regarding patient demographics (including age, sex, education, employment status, and geographic location), symptoms, underlying health conditions, and radiographic findings; at hospital discharge or end of the OPD visit, staff recorded diagnoses as assigned by a clinician in the medical record. Follow‐up data on patient outcomes were collected 30 days after hospital admission or OPD visit. Written, informed consent/assent was obtained from each participant, next of kin, and, in the case of minors, from the parent or primary caretaker before any evaluation activities were performed.

For this analysis, we compared characteristics of the patients enrolled in OPD (non‐hospitalized) to those who were enrolled in IPD (hospitalized). Because statistical tests can yield significant *p* values for small differences in large samples that are not clinically important, we used standardized mean difference (SMD) to measure the effect size between groups [11]. For categorical variables, SMD was calculated by dividing the difference in proportions by the pooled standard deviation [12]. An SMD < 0.2 was considered to indicate a negligible difference in variable distributions. All analyses were performed in R version 4.4.0.

The COVID‐19 vaccine effectiveness evaluation was approved by the WHO Regional Office for Africa Ethics Review Committee, the Scientific and Ethical Review Unit at the Kenya Medical Research Institute, and the Kenyan National Commission for Science, Technology, and Innovation. The protocol also received further administrative approval from each of the 20 enrolling sentinel health facilities in Kenya. Additionally, the protocol was reviewed by the U.S. Centers for Disease Control and Prevention (CDC), deemed not research, and approved to be conducted consistent with applicable federal law and CDC policy. Reliance was also obtained from the Washington State University Institutional Review Board.

## 3 Results

**FIGURE 1:**
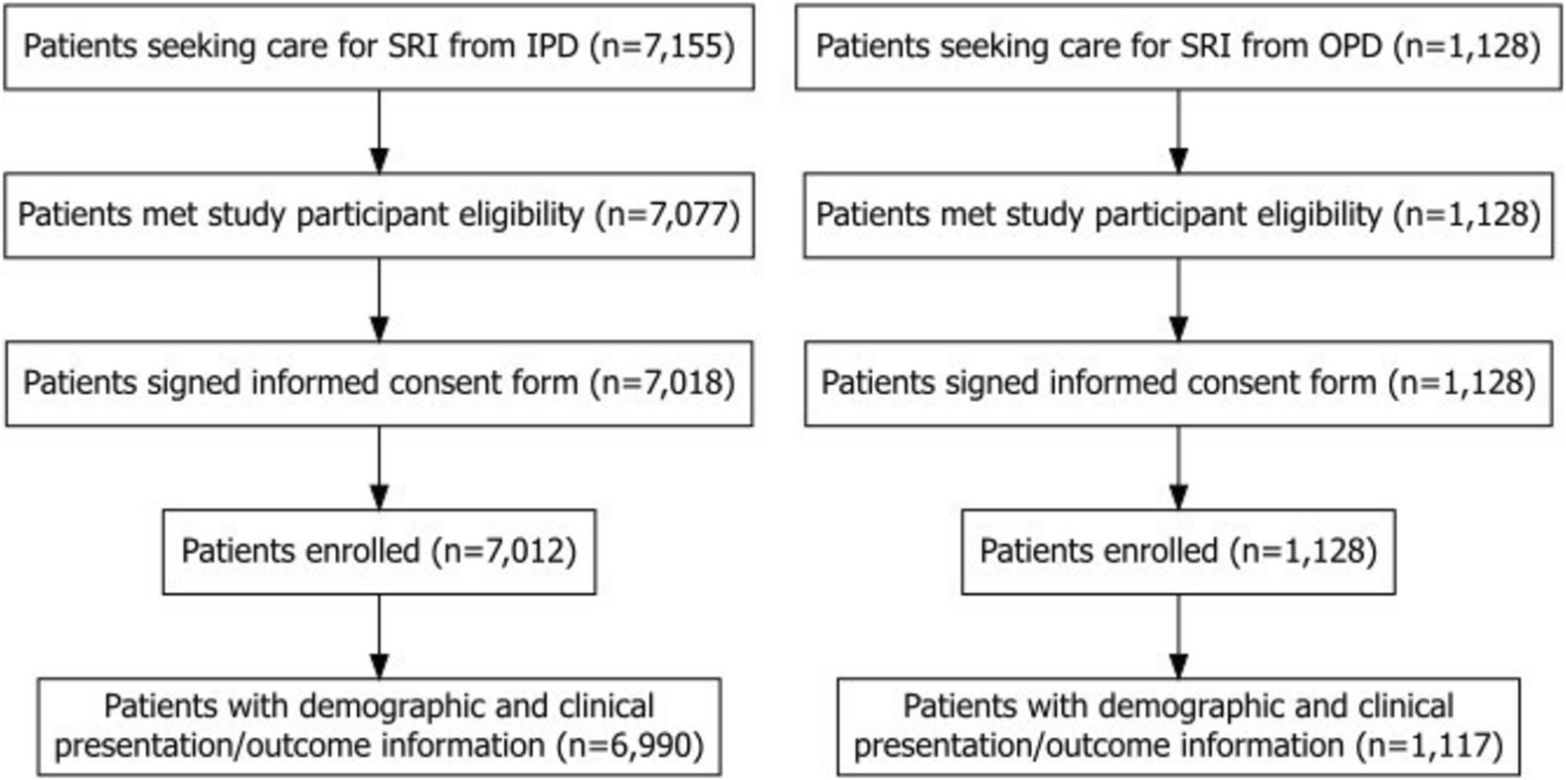
Flow diagram of patients included in the severe acute respiratory illness analysis of characteristics by hospitalization status—Kenya, 2022–2023. Abbreviations: IPD, inpatient department; OPD, outpatient department; SRI, severe respiratory illness. Footnote: To be included in the analysis, patients were required to have complete data on basic demographic characteristics (sex, age, occupation, and education), clinical symptoms and hospitalization history, and discharge diagnoses.

**FIGURE 2:**
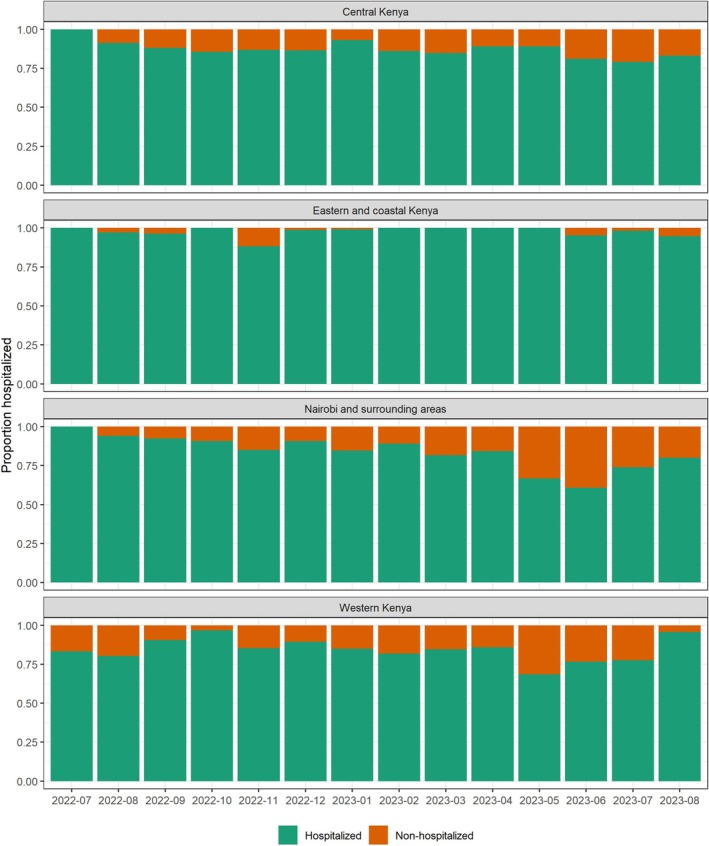
Proportion of hospitalized/non‐hospitalized patients enrolled, by month and geography—Kenya, 2022–2023. Footnote: Central Kenya included Nakuru, Nyahururu, Nyeri, Embu, and Meru county referral hospitals and Naivasha sub‐county hospital; Eastern and coastal Kenya included Machakos and Makueni county referral hospitals and Coast General Hospital; Nairobi and surrounding areas included Thika, Mbagathi, and Kiambu county referral hospitals, Kenyatta National Hospital, and Mama Lucy Hospital; and Western Kenya included Siaya, Migori, Kakamega, Kisumu, and Busia county referral hospitals and Jaramogi Oginga Odinga Teaching and Referral Hospital (JOOTRH).

During July 2022–August 2023, 8283 patients meeting the SRI case definition were screened at participating health facilities; of these, 8140 (98%) were enrolled (Figure 1). Of these, 8107 (>99%) patients had complete data on basic demographic characteristics (sex, age, occupation, and education), clinical symptoms and hospitalization history, and discharge diagnoses and were included in this analysis. This included 6990 (86%) hospitalized and 1117 (14%) non‐hospitalized patients. Both fever and cough, as commonly used in SARI case definitions [1], were reported by 1442 (20.6%) hospitalized and 270 (24.2%) non‐hospitalized patients. The proportion of non‐hospitalized patients enrolled by month varied slightly by region (Figure 2).

Compared with non‐hospitalized patients, hospitalized patients were more frequently >65 years (28% hospitalized versus 12% non‐hospitalized; SMD = 0.432) and without formal education (16% versus 7%; SMD = 0.649) (Table 1). Additionally, hospitalized patients less frequently reported formal employment (8% versus 22%; SMD = 0.398) compared with non‐hospitalized patients. Hospitalized patients also more commonly reported a diagnosis of an underlying health condition (61% versus 42%; SMD = 0.452) or a prior hospitalization in the previous 12 months (17% versus 8%; SMD = 0.271) than non‐hospitalized patients.

**TABLE 1:** Demographic characteristics and underlying health conditions of enrolled patients, by hospitalization status—Kenya, 2022–2023.

	Hospitalization status		
	Hospitalized	Non‐hospitalized^a^	SMD
	*N* = 6990	*N* = 1117	
	*n* (col%)	*n* (col%)	
Sex			0.110
Male	3770 (53.9%)	541 (48.4%)	
Female	3220 (46.1%)	576 (51.6%)	
Age (years)			0.432
<25	806 (11.5%)	244 (21.8%)	
25–64	4222 (60.4%)	728 (65.2%)	
65+	1962 (28.1%)	145 (13.0%)	
Education			0.649
No formal education	1099 (15.7%)	83 (7.4%)	
Primary	3302 (47.2%)	316 (28.3%)	
Secondary	1972 (28.2%)	398 (35.6%)	
Tertiary	617 (8.8%)	320 (28.6%)	
Occupation			0.398
Formal employment	549 (7.9%)	241 (21.6%)	
Self‐employed	2425 (34.7%)	307 (27.5%)	
Unemployed	4016 (57.5%)	569 (50.9%)	
Home‐health facility proximity^b^			0.071
Same subcounty	1991 (28.5%)	298 (26.7%)	
Different subcounty	4616 (66.0%)	809 (72.4%)	
Unknown	383 (5.5%)	10 (0.9%)	
Any chronic condition at enrollment^c^			0.452
Yes	4254 (60.9%)	467 (41.8%)	
No	2303 (32.9%)	622 (55.7%)	
Unknown	433 (6.2%)	28 (2.5%)	
Hospitalized in the previous 12 months			0.271
Yes	1202 (17.2%)	92 (8.2%)	
No	5788 (82.8%)	1025 (91.8%)	

*Note:* An SMD of < 0.2 is interpreted as indicating that the difference between groups is negligible.

Abbreviation: SMD, standardized mean difference.

^a^ Non‐hospitalized patients were those enrolled in outpatient departments, who were recommended for hospitalization by a clinician but were not hospitalized.

^b^ Patient's residential subcounty compared to the subcounty of the enrolling health facility.

^c^ Included ≥ 1 of the following conditions previously diagnosed by a clinician: chronic neurological or neuromuscular disease, tuberculosis, HIV/AIDS, chronic heart disease, malnutrition, chronic liver disease, chronic renal disease, diabetes, hypertension, asthma, cancer, sickle cell disease, or rickets.

**FIGURE 3:**
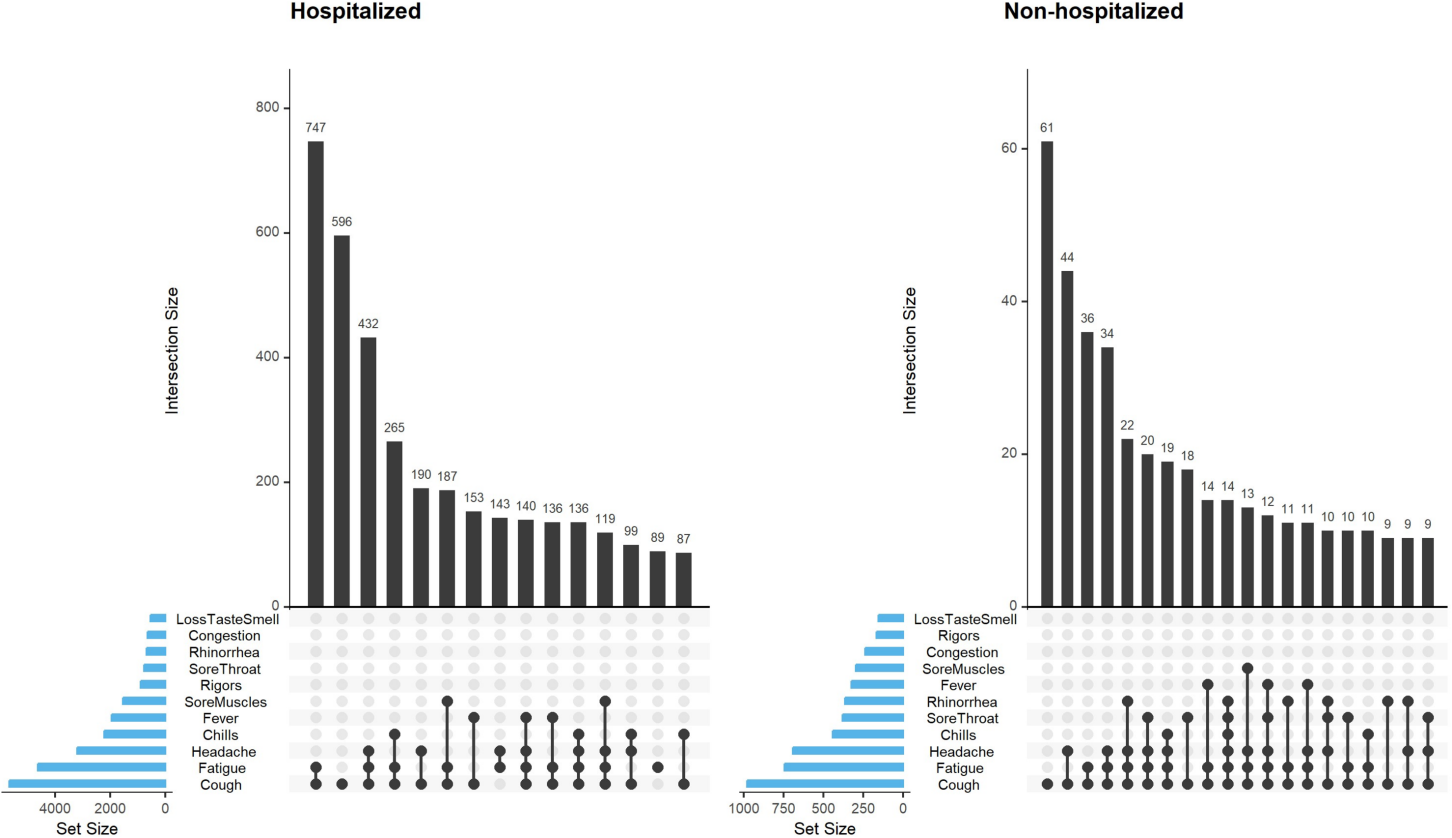
Symptomatology of enrolled patients, by hospitalization status—Kenya, 2022–2023. Footnote: Histograms (upper) display the frequency of the most common symptom combinations (lower) reported by patients at enrollment. Patients were enrolled if they met the following case definition: hospitalization or a clinician's recommendation for hospitalization for acute respiratory illness (onset within the last 14 days) and meeting one of the following clinical criteria: (1) at least two compatible symptoms, defined as cough, fever (reported or measured with a temperature of ≥38 °C), chills, rigors, myalgia, headache, sore throat, fatigue, nasal congestion, runny nose, or loss of taste or smell; or (2) pneumonia (based on clinical diagnosis or radiographic evidence).

Symptoms reported by both hospitalized and non‐hospitalized patients were similar, with cough, fatigue, and headache being the most common symptoms, either alone or in combination (Figure 3). A similar proportion of patients in both groups were found to have abnormal lung radiograph findings at enrollment (46% hospitalized, 41% non‐hospitalized; SMD = 0.089), and pneumonia was the most common discharge diagnosis assigned in both groups (55% hospitalized, 64% non‐hospitalized; SMD = 0.173) (Table 2). Patients in both groups were diagnosed with severe findings, with acute respiratory distress syndrome more common among hospitalized patients (12% versus 4%; SMD = 0.294) and sepsis diagnosed in 10% of patients in both groups (SMD = 0.001).

**TABLE 2:** Radiographic findings and discharge diagnoses^a^ assigned to enrolled patients, by hospitalization status—Kenya, 2022–2023.

	Hospitalization status		
	Hospitalized	Non‐hospitalized^b^	SMD
	*N* = 6990	*N* = 1117	
	*n* (col%)	*n* (col%)	
Abnormal lung radiograph findings			0.089
Yes	3242 (46.4%)	457 (40.9%)	
No	3191 (45.7%)	537 (48.1%)	
Unknown	557 (8.0%)	123 (11.0%)	
Discharge diagnoses^c^			
Pneumonia	3856 (55.2%)	711 (63.7%)	0.173
Hypertension	1352 (19.3%)	166 (14.9%)	0.119
Tuberculosis	1398 (20.0%)	89 (8.0%)	0.352
HIV/AIDS	1241 (17.8%)	81 (7.3%)	0.322
Anemia	909 (13.0%)	38 (3.4%)	0.355
Acute respiratory distress syndrome	831 (11.9%)	45 (4.0%)	0.294
Sepsis	681 (9.7%)	109 (9.8%)	0.001
Diabetes	722 (10.3%)	43 (3.8%)	0.255
Malaria	401 (5.7%)	119 (10.7%)	0.180
COVID‐19	357 (5.1%)	63 (5.6%)	0.024

*Note:* An SMD of < 0.2 is interpreted as indicating that the difference between groups is negligible.

Abbreviation: SMD, standardized mean difference.

^a^ Lung radiographs were taken at enrollment; discharge diagnoses were assigned at hospital discharge (hospitalized) or the end of the outpatient clinic visit (non‐hospitalized).

^b^ Non‐hospitalized patients were those enrolled in outpatient departments who were recommended for hospitalization by a clinician but were not hospitalized.

^c^ The 10 most assigned diagnoses are shown. More than one diagnosis could be assigned per patient. Some diagnoses might reflect underlying health conditions rather than acute incident conditions at the time of hospitalization. Diagnoses were abstracted as recorded by a clinician in the medical record; definitions and clinical criteria could have varied.

Of the 6990 hospitalized patients, 5355 (76.6%) reported their recovery status 30 days after admission; of these, 3547 (66%) reported full recovery. Among the 1808 hospitalized patients who had not fully recovered after 30 days, 102 (5.6%) were reported to have died. Of the 1117 non‐hospitalized patients, 1086 (97%) reported recovery status 30 days after their outpatient visit; of these, 841 (77.4%) reported full recovery. Among the 245 non‐hospitalized patients who had not fully recovered, 10 (4.1%) were reported to have died. Additionally, among the 245 non‐hospitalized patients who had not fully recovered, 74 (30.2%) sought care at another health facility, whereas 163 (66.5%) did not seek further care, and 8 (3.3%) had missing data. Of those who visited another health facility, 32 patients (44.4%) required hospitalization.

## 4 Discussion

Among patients enrolled in this COVID‐19 vaccine effectiveness evaluation in Kenya, we observed that non‐hospitalized patients (those who were referred for hospitalization but not hospitalized) had similar clinical severity to hospitalized patients; both groups had similar reported symptomatology and comparable levels of both sepsis diagnoses and abnormal lung radiographic findings at enrollment. In fact, more than 10% of non‐hospitalized patients were diagnosed with potentially life‐threatening conditions such as sepsis and acute respiratory distress syndrome, which can require intensive care [13, 14], underscoring that relying on data from hospitalized patients might underestimate the severity of respiratory illnesses. These findings emphasize the importance of including non‐hospitalized, severe cases in burden of disease estimation for respiratory pathogens. Additionally, patients in both the hospitalized and non‐hospitalized groups reported prolonged illnesses lasting for more than 30 days, highlighting the potential for these illnesses to cause lengthy health and economic impacts.

This analysis is subject to several notable limitations. First, the numbers of hospitalized and non‐hospitalized patients captured in this evaluation should not be used to infer the comparative incidence of hospitalized and non‐hospitalized severe respiratory illnesses; although this evaluation captured fewer patients in the OPD clinics than IPD wards, prior analyses have in fact indicated that the total incidence of non‐hospitalized severe respiratory illness might be greater than the incidence of hospitalized illness in Kenya, when including non‐medically attended cases [6, 7, 8]. Patients were enrolled in this evaluation by convenience sampling, and thus, the greater numbers of hospitalized patients might reflect ease of access to patients in IPD by enrollment staff, lower rates of refusal in IPD, or other factors. It is also possible that some patients who declined hospitalization and were characterized as OPD were hospitalized later in time or at another facility; however, based on available data, this proportion was low (32/1086; 3%). This analysis used an expanded case definition that included symptoms beyond cough and fever as used in standard SARI case definitions [1], which might limit comparability to SARI surveillance outcomes. It is possible that inclusion of additional non‐respiratory symptoms (chills, rigors, myalgia, headache, fatigue) in the case definition could have decreased the specificity of the case definition and captured cases caused by non‐respiratory pathogens; however, because the case definition required hospitalization or referral for an acute respiratory illness, this is unlikely. Additionally, we did not identify etiologies of severe respiratory illness among the hospitalized and non‐hospitalized patients that we compared. Furthermore, the analysis included patients aged ≥12 years, and results might not be generalizable to younger children. The results might also have limited generalizability to settings outside of Kenya, as health‐seeking behaviors and access to healthcare can differ, especially in high‐resource contexts.

Furthermore, some patients had missing data on variables such as location of residence, underlying health conditions, and abnormal lung radiograph findings; SMD calculations did not include the missing data and should be interpreted with caution. Additionally, this evaluation did not collect any qualitative data on the reasons for choosing or declining to be hospitalized following a clinician's recommendation; additional behavioral science evaluations are needed to characterize motivators/barriers for patients' decisions about hospitalization. Finally, this evaluation only characterized patients with severe respiratory illness who sought care at either IPD or OPD sections of a healthcare facility; there might be a substantial additional burden of severe respiratory illness among persons who did not or were not able to seek care [8], which warrants further evaluation.

In conclusion, these data underscore the importance of accounting for non‐hospitalized severe respiratory illnesses when estimating the full burden of disease for respiratory viruses. Because our evaluation used an expanded case definition for severe respiratory illness, we were able to examine non‐hospitalized illnesses among patients aged ≥12 years, highlighting the potential utility of expanded respiratory surveillance beyond SARI case definitions that capture only hospitalizations, primarily among children [5]. Our findings suggest that non‐hospitalized patients can have severe, even critical, respiratory illness, and thus indicate that the requirement of hospitalization as part of case definitions for severe illness might underestimate cases in resource‐constrained settings, such as in Kenya. Burden of disease estimates should continue to utilize multipliers from healthcare utilization surveys and other local/regional evidence to better account for severe respiratory illnesses associated with respiratory viruses among persons who would not be captured using standard hospital‐based surveillance [15, 16, 17, 18].

## Author Contributions

**Radhika Gharpure:** conceptualization, methodology, investigation, formal analysis, writing – original draft. **Young M. Yoo:** conceptualization, methodology, data curation, formal analysis, visualization, validation, writing – original draft. **Bryan O. Nyawanda:** conceptualization, methodology, data curation, investigation, validation, writing – review and editing. **Raphael O. Anyango:** investigation, writing – review and editing. **Brian O. Onyando:** investigation, writing – review and editing. **Sidney Ogolla:** investigation, writing – review and editing. **Billy Ogwel:** investigation, writing – review and editing. **Eric Osoro:** investigation, supervision, funding acquisition, writing – review and editing. **Philip Ngere:** investigation, writing – review and editing. **Samuel Kadivane:** investigation, writing – review and editing. **Nzisa Liku:** investigation, writing – review and editing. **Eva Leidman:** writing – review and editing. **Gideon O. Emukule:** conceptualization, methodology, investigation, supervision, funding acquisition, writing – review and editing. **Richard Omore:** conceptualization, methodology, investigation, supervision, funding acquisition, writing – review and editing. **Kathryn E. Lafond:** conceptualization, methodology, investigation, supervision, funding acquisition, writing – review and editing.

## Conflicts of Interest

The authors declare the following financial interests/personal relationships which may be considered as potential competing interests: Radhika Gharpure reports a relationship with Moderna, Inc. that includesemployment. During data collection, analysis, and write‐up, RG was employed by CDC.

## Disclaimer

The findings and conclusions in this report are those of the authors and do not necessarily represent the official position of the U.S. Centers for Disease Control and Prevention.

## Data Availability

Data available on request from the authors upon reasonable request.
